# Traditional uses, botanical description, phytochemistry, and pharmacological activities of *Phytolacca acinosa*: a review

**DOI:** 10.3389/fphar.2024.1480034

**Published:** 2025-01-09

**Authors:** Tooba Khalid, Chieh-Wei Chang, Samir Anis Ross, Faiza Naseer, Abdul Qadeer, Chien-Chin Chen, Hafiz Abdul Rafey

**Affiliations:** ^1^ Faculty of Pharmaceutical and Allied Health Sciences, Shifa college of Pharmaceutical Sciences (SCPS), Shifa Tameer-e-Millat University, Islamabad, Pakistan; ^2^ Division of General Surgery, Department of Surgery, Ditmanson Medical Foundation Chia-Yi Christian Hospital, Chiayi, Taiwan; ^3^ The National Center for Natural Products Research, Bio Molecular Science Department, Division of Pharmacognosy, University of Mississippi, Oxford, MS, United States; ^4^ Department of Cell Biology, School of Life Sciences, Central South University, Changsha, China; ^5^ Department of Pathology, Ditmanson Medical Foundation Chia-Yi Christian Hospital, Chiayi, China; ^6^ Department of Cosmetic Science, Chia Nan University of Pharmacy and Science, Tainan, Taiwan; ^7^ Doctoral Program in Translational Medicine, National Chung Hsing University, Taichung, Taiwan; ^8^ Department of Biotechnology and Bioindustry Sciences, College of Bioscience and Biotechnology, National Cheng Kung University, Tainan, Taiwan

**Keywords:** *Phytolacca acinosa*, pharmacological activity, medicinal plant, phytochemistry, bioactive compounds, traditional use

## Abstract

**Background:**

*Phytolacca acinosa* is an herbaceous herb belonging to the Phytolaccaceae family. The plant has a long history of usage in traditional medicine for treating a variety of ailments including infectious diseases, edema, inflammation, gastric, and abdominal distress. The traditional use, phytochemistry, and pharmacological properties of *Phytolacca acinosa* are outlined in this article.

**Main text:**

To date, few bioactive molecules have been identified and isolated from the plant, such as phytolacacinoside A, esculentoside H, jaligonic acid and esculentoside B, phytolaccanol and epiacetyl aleuritolic acid, esculentoside A, esculentoside C, esculentoside D, esculentoside T, esculentoside S, sitosterol. The literature related some of the reported ethnomedicinal uses of the plant to these compounds found in different parts of the plant.

**Conclusion:**

The in-depth knowledge about the significance of *Phytolacca acinosa* presented in this review may open up opportunities for research development in drug discovery and a better comprehension of the therapeutic advantages of the plant.

## Background

Humans have relied heavily on plants for sustenance and disease treatment for many years. According to estimates, 75% of the world’s population relies on herbal remedies for their essential medical requirements (Ahmad et al., 2021). In fact, a lot of the medications used in contemporary medicine today are derived from plants. Research to examine the active medicinal components, effectiveness, and safety of such plants has increased due to their widespread usage in traditional medicine. According to the literature, the search for novel therapeutic compounds based on folklore knowledge about medicinal plants and traditional applications learned from folklore usage may serve as a reliable method for the creation of new therapeutic compounds. To strengthen the scientific case for their therapeutic properties and to confirm the safety of their usage in traditional medicines, data and high-quality research on medicinal plants are required (Tekuri et al., 2019).

Around the world, traditional medicine is still practiced today. It has become increasingly important economically, especially with the use of medicinal plants that are still in high regard, in developing nations where access to modern healthcare is limited and traditional medicine is the only available treatment. The World Health Organization reports that current estimates indicate that a significant portion of the population in many affluent nations engages in some form of traditional practice, particularly the use of medicinal plants. For historical and cultural reasons, the use of therapeutic herbs has remained popular even if access to modern treatment is not an issue in many countries. In terms of biodiversity, conservation, and sustainable use, medicinal plants form a significant economic and health component (Sun and Shahrajabian, 2023). Deeper insights into finding bioactive products that can be used as anti-infective agents will depend much on information about the traditional knowledge of medicinal plants and their usage (Alam et al., 2021).

According to the literature, the search for novel therapeutic compounds based on folklore knowledge about medicinal plants and traditional applications learned from locals may serve as a technique for the creation of new therapeutic compounds. To strengthen the scientific case for their therapeutic properties and to confirm the safety of their usage in traditional medicines, data and high-quality research on medicinal plants are required. In order to provide thorough information about the traditional uses, pharmacological activities, phytochemical components, and isolated compounds of the plant and to encourage additional research for its potential use either as an isolated or in polyherbal formulations and to serve as the lead for the novel therapeutic agents, we reviewed the literature. To our knowledge, this draft is the first review study that provides comprehensive information about Phytolacca *acinosa.*

## Methodology

**FIGURE 1:**
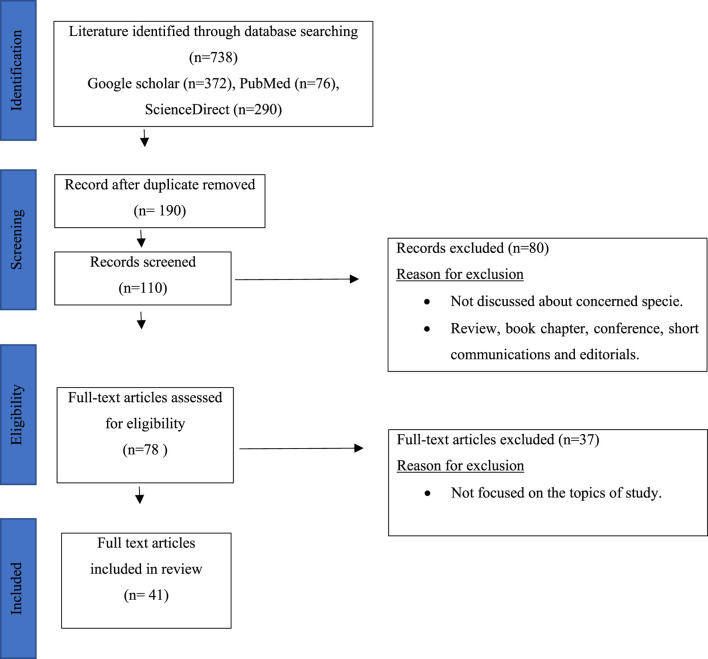
Inclusion and exclusion criteria.

Research papers related to *Phytolacca acinosa* were thoroughly reviewed using various websites (Google scholar, PubMed, and ScienceDirect) and keywords (Phytochemistry, traditional uses, Pharmacological uses, and *Phytolacca acinosa*), as shown in Figure 1. The number of articles on *Phytolacca acinosa* was 738, and after removal of duplicate articles and articles not related to the topic, 41 articles were left. These articles were concerned about isolation of metabolites, traditional uses, and pharmacological uses of *Phytolacca acinosa.* Various bioactive metabolites were isolated from *Phytolacca acinosa,* which can be used as therapeutic agents for various diseases. These metabolites are promising candidates in the pharmaceutical industry for the development of novel medications and future clinical uses. In total, 41 publications describing the compound isolation and the biological activities of the extracts were used, excluding articles merely on botany and/or agronomy. The isolated components were categorized by class and part mentioned Table 2 to discriminate between publications describing metabolite isolation (including NMR data) or screening (by means of TLC and LC). The reported pharmacological activities of crude and fractionated extracts were categorized by species in Table 3. The biological activities of the isolated compounds as well as fractions were also taken into account. Plant taxonomy was validated using the databases of International Plant Names Index (IPNI), International Plant Names Index (IPNI), and Med PlantBas.

### Botanical description of *Phytolacca acinosa*

The flowering plant *Phytolacca acinosa*, known as Indian pokeweed, Sarangum (Ijaz et al., 2019), Rinsag, Lubar, Yeluobo, Sweet Belladonna, and Matazor belongs to the Phytolaccaceae family (Saleri et al., 2017). *Pircunia latbenia* (Moq.) and *Phytolacca latbenia* (Moq.) Walter are synonyms of *Phytolacca acinosa*. It was widely introduced to Europe and is indigenous to temperate eastern Asia, including the Himalayas, the majority of China, Vietnam, and Japan (Pliszko and Klich, 2018). Reviewing the available literatures, there is no comprehensive and detailed review concerning the plant’s pharmacological effects, phytochemical composition, and traditional usage. Phytolacca is a genus of perennial plants indigenous to East Asia, South America, and North America. This genus belongs to the family Phytolaccaceae. In both Asian and Western traditional medicine, plants from the Phytolaccaceae family are frequently used to treat inflammation, edema, dermatitis, tumors, rheumatism, bronchitis, and molluscicides (Saleri et al., 2017). Triterpene-containing saponins account for the majority of the chemical components in these plants, along with flavonoids, triterpenes, phenolic acids, polysaccharide, and sterols (Sydor et al., 2022). It has 25–35 species worldwide, including four species in the People’s Republic of China (Song et al., 2022). Species of this genus accepted by one or more regional flora includes *Phytolacca acinosa* Roxb., *Phytolacca americana* L., *Phytolacca polyandra* Batalin, *Phytolacca australis* Phil, *Phytolacca pruinosa* Fenzl, *Phytolacca bogotensis* Kunth, *Phytolacca rivinoides* Kunth and C.D.Bouché*, Phytolacca chilensis* Miers, *Phytolacca dioica* L., *Phytolacca sandwicensis* Endl., *Phytolacca dodecandra* L'Hér., *Phytolacca heterotepala* H. Walt., *Phytolacca icosandra* L., *Phytolacca japonica* Makino, *Phytolacca thyrsiflora* Fenzl ex J.A.Schmidt, *Phytolacca octandra* L., and *Phytolacca weberbaueri* H. Walt. We are focusing in this review on *Phytolacca acinosa* (one of the specie of genus Phytolacca).

**FIGURE 2:**
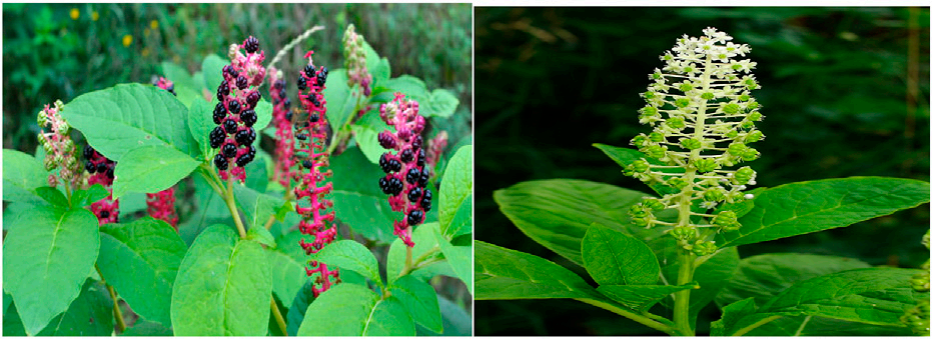
*Phytolacca acinosa* plant view.

*Phytolacca acinosa* is a perineal angiosperm found predominantly in subtropical region. It is usually an herbaceous shrub, up to 1.5 m height, with white flowers (Balogh and Juhász, 2008), as shown in Figure 2. Leaves are elliptical with papery texture. The stem is fleshy, green, or reddish purple in color with longitudinal grooves. Its fruit looks like small berries, while roots are thick and fleshy. This weed is frequently found in hillsides, roadsides, valleys, cultivated ground, damp wayside areas, and also growing inside forests and forest margins at the elevation of 500–3,400 m (Magray et al., 2022). It is used as a herb and spice in food. Young leaves are used as a vegetable in cooking.

### Traditional uses of *Phytolacca acinosa*

Several traditional remedies, including Chinese, Tibetan, and other Asian folk medicines, use the plant *Phytolacca acinosa* Roxburgh. Traditional Chinese medicine has long utilized *Phytolacca acinosa* Roxb., which is included in the Chinese Pharmacopoeia, as a diuretic and also to treat a variety of ailments such edema, swelling, and blisters (Ma et al., 2017). The herb has narcotic, anti-inflammatory, and antiviral properties (Bown, 1995). The oil from the root is used for pain in joints (Dhar et al., 1977). Roots are used as an antidote, vermifuge, and also taken to alleviate cough, constipation, urinary issues, infectious diseases, and abdominal distension (Bown, 1995; Duke and Ayensu, 1985). They are also used to treat boils and ulcers (Bown, 1995). In India, *Phytolacca acinosa* (known as “jagra”) fresh leaves are boiled, mashed, and fried in cooking oil with spices (Bailly, 2021). Fruits can be used to add flavor in food. Fatty oil comes from their seeds (Sharma et al., 2010). The summary of traditional uses and their pharmacological actions of different parts of *Phytolacca acinosa* along with scientific evidence is presented in Table 1.

**TABLE 1:** Traditional uses of different parts of *Phytolacca acinosa* and scientific confirmation of their pharmacological activity.

Traditional uses	Plant part	References	Confirmation of pharmacological activity
Cough and constipation	Roots	Bown (1995); Duke and Ayensu (1985)	Not confirmed
Infectious diseases	Roots	Bown (1995); Duke and Ayensu (1985)	Confirmed (GuoDong et al., 2010; Ma et al., 2019)
Ulcers	Roots	Bown (1995)	Confirmed (Li et al., 2022; Ma et al., 2019)
Inflammation	Not specified	Bown (1995)	Confirmed (Koul et al., 2003; Wei et al., 2015)
Pain	Roots	Dhar et al. (1977)	Not confirmed
Edema, blisters, swelling, and diuretic	Roots	Ma et al. (2017)	Not confirmed
Antidote and vermifuge	Roots	Bown (1995); Duke and Ayensu (1985)	Not confirmed
Urinary issues	Roots	Bown (1995)	Not confirmed
Abdominal distension	Roots	Duke and Ayensu (1985)	Not confirmed
Narcotic	Not specified	Bown (1995)	Not confirmed

### Phytochemistry of *Phytolacca acinosa*

The presence of phytochemical components (secondary metabolites) and other bioactive substances, which aid in the discovery and development of new therapeutic agents, is crucial for the pharmacological activities of traditionally used plants. Some of the phytochemical constituents include glycosides, alkaloids, proanthocyanidins, flavonoids, tannins, phenylpropanoids, terpenoids, resins, furocoumarins, lignans, proteins, naphthodianthrones, and peptides (Senthilkumar et al., 2018). Their safety and effectiveness increase the industrial and pharmaceutical use of compounds isolated from medicinal plants (Ahmad et al., 2021).

*Phytolacca acinosa* is rich in different phytochemicals including triterpenoid saponins, triterpenoid glycosides, glycosides, olenane derivatives, phenols, flavones, phytosterols, and polyphenolic compounds (Du et al., 2018; Gao et al., 2009; Ijaz et al., 2019; Li et al., 2022; Ma et al., 2019; Razdan et al., 1983).

### Identified bioactive metabolites from *Phytolacca acinosa*

In a research study, 75% ethanolic extract of the root of *Phytolacca acinosa* Roxb was used for isolation of phytolacacinoside A (a novel triterpenoid saponin), along with some known compounds. Physicochemical properties and spectroscopic data were used for their structural elucidation, and on the basis of their analysis, the following compounds were isolated: daucosterol, 3-*O*-β-[(β-d-glucopyranosyl-(1 → 4)-*O*-β-d-xylopyranosyl)]-11β-methoxy-jaligonic acid 30-methyl ester 28-*O*-β-d-glucopyranoside, 3-*O*-β-[(β-d-glucopyranosyl-(1 → 4)-*O*-β-d-xylopyranosyl)]-jaligonic acid 30-methyl ester (phytolaccoside E), 3-*O*-β-[(β-d-glucopyranosyl-(1 → 4)-*O*-β-d-xylopyranosyl)]-jaligonic acid 30-methyl ester 28-*O*-β-d-glucopyranoside (esculentoside G), 3-*O*-β-d-xylopyranosyl-jaligonic acid 30-methyl ester (phytolaccoside B), palmitic acid monoglyceride, hypaphorine, and β-sitosterol (Gao et al., 2009).

A study demonstrated that the defatted alcoholic extract of *Phytolacca acinosa* is rich in various components. The defatted alcoholic extract of *Phytolacca acinosa* was used for isolation and characterization of 30-methylspergulagenate along with three new oleanane derivatives like isophytolaccinic acid A, spergulagenic acid A, and isophytolaccagenin A. The new identified compounds are 3β,23α-diacetoxy-28β-methyloleanate-12-en-30β-oic acid, 3β-acetoxy-30β-methyloleanate-12-en-28β-oic acid and 2β, and 3β,23α-triacetoxy-28β-methyloleanate-12-en-30β-oic acid (Razdan et al., 1983).

Strauss et al., 1995 reported the presence of triterpenes in *Phytolacca acinosa* root cultures, such as esculentoside B, S, A, H, phytolaccoside F (3-O-α-l-rhamnopyranosyl-(1 → 2)-β-d-glucopyranosyl-(1 → 2)-β-d-xylopyranosyl-phytolaccagenic acid), esculentoside L_1_ (3-O-β-d-glucopyranosyl-(1 → 2)-β-d-xylopyranosyl-28-O-β-d-glucopyranosyl-phytolaccagenic acid), and esculentoside R, identified by TLC, ^13^C, ^1^H NMR, and mass spectra.

Another study was conducted in which a new glycoside named as esculentoside S has been identified as 3-O-β-D-xylopyranosyl-28-O-β-D-glucopyranosyl-phytolaccagenin from the leaves of *Phytolacca acinosa* along with two known glycosides, esculentoside B and esculentoside A (Spengel and Schaffner, 1993).

A group of researchers isolated a new triterpenoid saponin named esculentoside T together with five already known compounds, namely, esculentoside A, esculentoside B, esculentoside C, esculentoside D, and esculentoside H, from the roots of *Phytolacca acinosa n*-butanol extract, and their structure elucidation was conducted by spectral data (He et al., 2011).

A group of researchers studied defatted ethanolic extract, and two novel triterpenoids, phytolaccanol and epiacetyl aleuritolic acid, as well as β-sitosterol have been extracted (Razdan et al., 1982).

A study isolated a new triterpenoid saponin (esculentoside U) from the *Phytolacca acinosa* root extract (Du et al., 2018).

A group of researchers isolated jaligonic acid B along with three known triterpenoids: esculentoside H, jaligonic acid, and esculentoside B from *Phytolacca acinosa* root extract. Spectroscopic techniques like MS, IR, and 1D and 2D NMR were used for their structural elucidation (Ma et al., 2019).

In a research study, the ethanolic extract of the root of *Phytolacca acinosa* was used for isolation purpose and two new flavones cochliophilin B, 6,7-methylenedioxy-4-hydroxypeltogynan-7′-one as well as two known ones, 6-methoxy-7-hydroxy flavone and cochliophilin A were isolated. IR, MS, and 1D- and 2D-NMR were used for their structure determination (Ma et al., 2017).

A study demonstrated isolation of compounds from the fruit of *Phytolacca acinosa* fermentation broth. Two new oleanane-type triterpene glycosides, phytolasides A and B and six known ones, include kaempferol, kaempferol-3-*O*-*β*-D-glucoside, kaempferol-3-*O*-*β*-D-glucose-(2→1)-*β*-D-apiose, mannitol, L-phenylalanine, and cyclo (L-pro-L-Tyr), were isolated. HR-ESI-MS and 1 D- and 2 D-NMR spectroscopic techniques were used for structural elucidation of these compounds (Li et al., 2022). Different compounds with their class and parts used are shown in Table 2.

**TABLE 2:** Isolated metabolites with their class and part used.

Metabolite name	Class	Type	Part used	References
Phytolacacinoside A (1)	Triterpenoid saponin	Cyclic compound	Root	Gao et al. (2009)
Esculentoside B (2)	Triterpenoid glycoside	Heterocyclic compound	Root Leaves	Abekura et al. (2019), He et al. (2011), Ma et al. (2019), Spengel and Schaffner (1993), Strauss et al. (1995), Tao et al. (2020)
Esculentoside S (3)	Glycoside	Heterocyclic compound	Leaves Root	Spengel and Schaffner (1993), Strauss et al. (1995), Zhao et al. (2021)
Esculentoside C (4) and D (5)	Triterpenoid saponin	Heterocyclic compounds	Root	He et al. (2011)
Cochliophilin A (6), B (7) and 6-methoxy-7-hydroxy flavone (8)	Flavones	Heterocyclic compounds	Root	Ma et al. (2017)
Acinospesigenin A (9), B (10) and C (11)	Triterpenoid	Cyclic compounds	Leaves Fruit	Koul et al. (2003), Spengel and Schaffner (1989)
Esculentoside A (12)	Triterpene saponin	Heterocyclic compound	Whole plant Root Leaves	He et al. (2011), LAI & SUN (2008), Spengel and Schaffner (1993), Strauss et al. (1995)
Esculentoside R (13)	Triterpene saponins	Heterocyclic compound	Root	Strauss et al. (1995), Zhao et al. (2021)
Ursolic acid (14)	Triterpenoid	Cyclic compound	Fruit	Dhar et al. (1977)
Isophytolaccinic acid A (15)	Olenane derivative	Cyclic compound	Fruit	Razdan et al. (1983)
Phytolaccanol (16)	Triterpenoids	Cyclic compound	Fruit	Razdan et al. (1982)
Phytolasides A (17) and B (18)	Triterpene glycosides	Cyclic compounds	Fruit	Li et al. (2022)
Phytolaccagenin (19) and esculentic acid (20)	Triterpenoids	Cyclic compounds	Leaves Fruit	Harkar et al. (1984), Spengel and Schaffner (1989)
Esculentoside G (21)	Triterpenoid saponin	Heterocyclic compound	Root	Gao et al. (2009), Zhao et al. (2021)
Esculentoside H (22)	Triterpenoid saponin	Heterocyclic compound	Root	He et al. (2011), Ma et al. (2019), Zhao et al. (2021)
Phytolaccoside B (23) and E (24)	Triterpenoids	Heterocyclic compounds	Root	Gao et al. (2009), Zhao et al. (2021)
Esculentoside T (25)	Triterpenoid saponin	Heterocyclic compound	Root	He et al. (2011), Zhao et al. (2021)
Esculentoside U (26)	Triterpenoid saponin	Heterocyclic compound	Root	Du et al. (2018), Ma et al. (2019), Zhao et al. (2021)
Esculentoside E (27), F (28), and L (29)	Triterpenoid saponin	Heterocyclic compounds	Root	Zhao et al. (2021)
Esculentoside L1 (30)	Triterpenoid saponin	Heterocyclic compound	Root	Strauss et al. (1995), Zhao et al. (2021)
Phytolaccoside F (31)	Triterpenoids	Heterocyclic compound	Root	Strauss et al. (1995), Zhao et al. (2021)
Jaligonic acid (32)	Triterpenoids	Cyclic compound	Root Fruit	Harkar et al. (1984), Ma et al. (2019)
β-sitosterol (33)	Phytosterols	Cyclic compound	Root Fruit	Gao et al. (2009), Razdan et al. (1982)
Daucosterol (34)	Steroid saponin	Heterocyclic compound	Root	Gao et al. (2009)
*Hypaphorine* (35)	Indole alkaloid	Heterocyclic compound	Root	Gao et al. (2009)
Spergulagenic acid A (36)	Triterpenoids	Cyclic compound	Berries	Razdan et al. (1983)
Phytolaccagenic acid (37)	Triterpenoids	Cyclic compound	Berries	Harkar et al. (1984)
Kaempferol (38) and kaempferol-3-*O*-*β*-D-glucoside (39)	Flavanol	Heterocyclic compounds	Fruit	Li et al. (2022)

**FIGURE 3:**
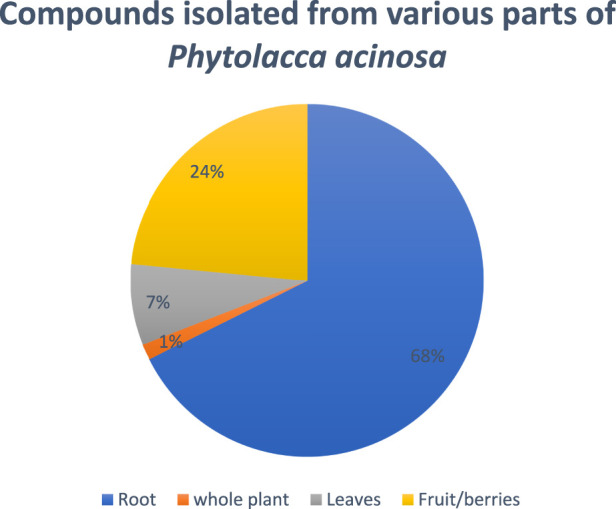
Percentage of isolated compounds from various parts of *Phytolacca acinosa*.

A research study was conducted on defatted berries of *Phytolacca acinosa,* which led to the isolation and characterization of five known triterpenoids, phytolaccagenin, acinosolic acid, phytolaccagenic acid, jaligonic acid, and esculentic acid, and three new oleanane derivatives named as acinosolic acid A, phytolaccagenin A, and acinosolic acid B. The new compounds have been identified as 3β-acetoxy-28β-methyloleanate-12-en-2β-ol-30β-oic acid, 3β-acetoxy-3β-methyloleanate-12-en-2β,23α-diol-28β-oic acid, and 2β-acetoxy-28β-methyloleanate-12-en-3β-ol-30β-oic acid (Harkar et al., 1984). Different compounds isolated from various parts of *Phytolacca acinosa* are shown in Figure 3. Most of the compounds were isolated from the root part of this plant.

In an experimental study, Koul et al. isolated three new triterpenoids from *Phytolacca acinosa* named as acinospesigenin B, A, and C and characterized as olean-12-en-23-aL-2β,3β-dihydroxy-30-methoxycarbonyl-28-oic acid, 3β-acetoxy-11α,23-dihydroxytaraxer-14-en-28-oic acid, olean-12-en-23-aL-2β, and olean-12-en-23-aL-2β,3β,11α-trihydroxy-30-methoxycarbonyl-28-oic acid, respectively (Koul et al., 2003).

Dhar et al. found that *Phytolacca acinosa* Roxb. fruit contains ursolic acid, ursolic acid galactoside, n-pentacosane, lignoceryl palmitate, 16-hentriacontanol, and myristic acid (Dhar et al., 1977).

Lai et al. found esculentoside A in different parts of *Phytolacca acinosa* Roxb. and *P. americana L.* Separation was performed by using the Diamonsil C18 column (5 µm, 250 mm × 4.6 mm) and methanol–water (75:25) as the mobile phase. The flow rate was 1 mL per minute. An evaporative light scattering detector was used for detection (Lai and Sun, 2008).

In an experimental study, phytolaccagenin and acinospesigenin were isolated from the leaves of *Phytolacca acinosa* (Spengel and Schaffner, 1989).

In another study, triterpene saponins isolated from the root of *Phytolacca acinosa Roxb* were analyzed using high-performance liquid chromatography coupled with electrospray ionization and quadrupole time-of-flight mass spectrometry (HPLC-ESI-QTOF-MS/MS). A total of 29 triterpene saponins were found, out of which 16 were already reported, esculentoside G, polyandrasides B, phytolaccasaponin N-5, esculentoside F, esculentoside H, esculentoside E, esculentoside R, esculentoside L1, esculentoside U, phytolaccoside E, esculentoside L, esculentoside S, esculentoside B, esculentoside T, phytolaccoside F, and phytolaccoside B, and 13 new compounds such as 3-O-(β-D-glucuronopyranoside)-28-Oβ-D-glucopyranosyl phytolaccagenin, 3-O-(β-D-glucuronopyranoside)-28-Oβ-D-glucopyranosyl-30- methyloleanate-9, 12-dien-2, 3, 23-trihydroxyl-28-oic acid, 3-O-[β-D-glucopyranosyl (1 → 4)- β-D-xylopyranosyl]-esculentic acid, 3-O-[α-L-rhamnopyranosyl-(1 → 2)-O-β-D-glucopy ranosyl-(1 → 2)-O-β-D-xylopyranosyl] -jaligonic acid 30-methyl ester, 3-O-[β-D-xylopyranosyl]- 28-O-β-D-glucopyranosyl-1 1-oxo-30-methyloleanate- 12-en- 2β,3β,23-trihydroxy-28-oic acid, 28-O-(β-D-xylopyranosyl]-11α-hydroxyphytolaccagenin, 3-O-[β-D-xylopyranosyl -(1 → 2)-β-D -xylopyranosyl)-28-O-β-D-glucopyranosyl phytolaccagenin, 3-O-(β-D-glucopyranosyl) serjanic acid, 3-O-[β-D-glucopyranosyl]-28-O-β-D-glucopyranosyl-phytolaccagenic acid, 3-O-[β-D-xylopyranosyl]- 11-oxo-30-methyloleanate- 12-en-2β, 3β,23-trihydroxy-28-oic acid, 3-O-[α-L-rhamnopyranosyl-(1 → 2)-O-β-D- glucopyranosyl-(1 → 2)-O-β-D-xylopyranosyl]-oleana−12-en-28, 30-dioic acid, 3-O-(β-D-xylopyranosyl)-28-O-β-D-glucopyranosyl-30-methyloleanate-9, 12-dien- 3, 23-dihydroxyl-28-oic acid, and 3-O-(O-α-L-rhamnopyranosyl-(1 → 2)- β-D-glucopyranosyl-(1 → 2)- β-D-xylopyranosyl) serjanic acid were detected. This study shows that the characterization of complex triterpene saponins in herbal extracts may be performed quickly and effectively using the HPLC-ESI-QTOF-MS/MS technique (Ling et al., 2022; Zhao et al., 2021).

**FIGURE 4:**
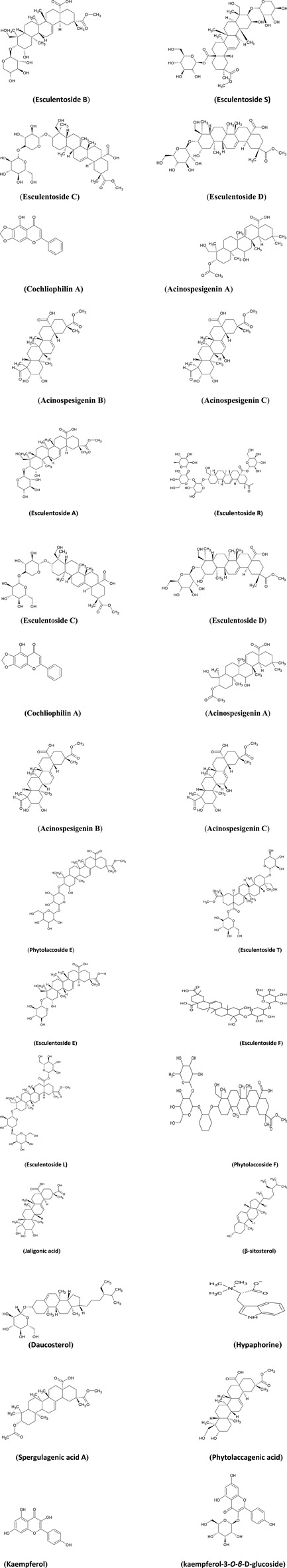
Chemical structure of bioactive metabolites from *Phytolacca acinosa*.

**FIGURE 5:**
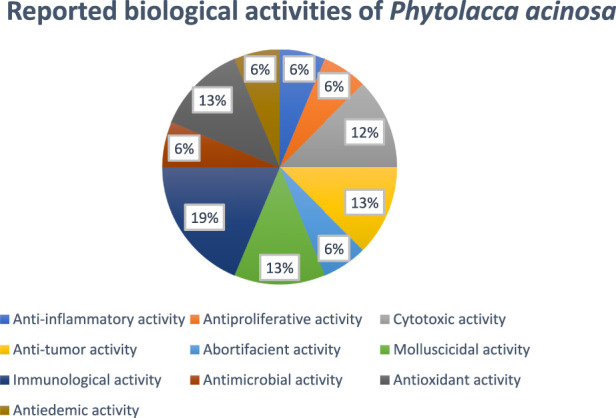
The percentage variations in the pharmacological evaluations for *Phytolacca acinosa*.

The chemical structures of bioactive compounds isolated from Phytolacca acinosa along with its various part's percentage contributing to the biological activities are presented in Figures 4, 5.

## Pharmacological activities of *Phytolacca acinosa*

### Anti-inflammatory activity

Abekura et al. assessed the anti-inflammatory activity of esculentoside B. From the n-butanol fraction of the aqueous alcoholic extract of the roots of *Phytolacca acinosa* Roxb, esculentoside B has been isolated and identified. First, they assessed esculentoside B’s cytotoxicity against RAW 264.7 macrophage cells. Different concentrations of esculentoside B (0, 10, 30, 50, 70, and 90 μM) or a combination of esculentoside B and lipopolysaccharides (LPS) were used to treat the cells. After 24 h into the procedure, the cytotoxic parameter of cell viability was evaluated periodically using MTT-based assay with the reagent. Esculentoside B therapy for RAW 264.7 cells was not cytotoxic at doses of 0–50 μM. These findings imply that esculentoside B at 50 μM has no cytotoxicity on the macrophage RAW 264.7 cells. Then, they investigated the anti-inflammatory activities of esculentoside B in LPS-treated macrophage RAW 264.7 cells and found that esculentoside B at concentration of (0, 30, and 50 μM) with or without 100 ng/mL of LPS inhibited production of nitric oxide (NO) and also downregulated gene and protein expression levels of cyclooxygenase-2 and inducible isoform of NO synthase (iNOS) in a dose-dependent manner. Nitric oxide amount in the medium was measured using Griess assays. Western blot analysis was used for assessing protein levels of COX-2 and iNOS. iNOS and COX-2 mRNA levels were determined by RT-PCR. Esculentoside B may also reduce inflammation by preventing nuclear factor kappa-B (NF-κB) intracellular translocation and the phosphorylation of the c-Jun N-terminal kinase (JNK) signal in macrophage RAW 264.7 cells. For determination of effects of EsB on LPS-stimulated nuclear translocation of NF-κB in macrophage RAW 264.7 cells, they were treated for 30 min at various concentrations of EsB (0, 30, and 50 μM) and then further incubated in the absence or presence of LPS (100 ng/mL) for 10 min. Cells were harvested to prepare nuclear and cytosolic extracts, and these extracts were separated by SDS-polyacrylamide gel electrophoresis for Western blot analysis. Lamin B and β-actin were used as internal controls. Confocal microscopy was used for nuclear translocation of NF-κB. For immunostaining of cells, fluorescein isothiocyanate (for NF-κB) and Hoechst (for nuclei) were used. RAW 264.7 cells were treated for 30 min with various concentrations of 0, 30, and 50 μM of EsB, followed by incubation with or without LPS (100 ng/mL) for 10 min. Phosphorylation levels of extracellular signal-regulated kinase ½ (ERK), p38, and JNK in protein samples were analyzed by Western blotting. ERK, JNK, and p38 were used as loading controls (Abekura et al., 2019).

### Anti-proliferative activity

Various metabolites were isolated from the *Phytolacca acinosa* fruit fermentation broth. *In vitro* anti-proliferative activity of these metabolites (phytolasides A, phytolasides B, kaempferol, kaempferol-3-*O*-*β*-D-glucoside, kaempferol-3-*O*-*β*-D-glucose-(2→1)-*β*-D-apiose, mannitol, L-phenylalanine, and cyclo (L-pro-L-Tyr)) was determined on hepatocellular carcinoma cell lines (HepG2) using Cell Counting Kit-8. Phytolasides A and B showed anti-proliferative effects on HepG2 cells by reducing the viable cell concentration. The anti-proliferative activity of phytolasides A and B was determined by the IC_50_ value of 12.524 ± 0.659 *μ*M and 14.738 ± 0.725 *μ*M, respectively. Doxorubicin was used as a positive control, which showed the IC_50_ values of 1.463 ± 0.059 lM (Li et al., 2022).

### Cytotoxic activity

Metabolites such as jaligonic acid B, esculentoside H, jaligonic acid, and esculentoside B isolated from the *Phytolacca acinosa* root extract were used for cytotoxicity assay, which was performed on three human cancer cell lines human leukemia HL-60 cell line, human hepatocellular carcinoma BEL-7402 cell line, and human liver cancer HepG_2.2.1.5_ cell line by the colorimetric MTT assay. Esculentoside B exhibited moderate cytotoxic activity against HL-60 (25.80 µM) and HepG_2.2.1.5_ (73.9 µM) and weak inhibitory activity against BEL-7402 (188.41 µM) (Ma et al., 2019).

Metabolites such as 6,7-methylenedioxy-4-hydroxypeltogynan-7′-one, cochliophilin A, cochliophilin B, and 6-methoxy-7-hydroxy flavone were isolated from the ethanolic extract of the root of *Phytolacca acinosa.* All the compounds were tested for their *in vitro* activity at concentrations ranging from 1 to 100 μmol/L against A549 (human lung cancer cell line), BEL-7402 (human hepatic carcinoma cell line), and HL-60 (human leukemia cell line). Cochliophilin B and 6-methoxy-7-hydroxy flavone exhibited moderate inhibition in the MTT assay against BEL-7402 (human hepatic carcinoma cell line), with the IC_50_ values of 28.22 and 39.16 μmol/L, respectively. Doxorubicin was used as the positive control with the IC_50_ value of 8.12 μmol/L. DMSO was used as the negative control (Ma et al., 2017).

### Anti-tumor activity

In one of the experimental studies, *Phytolacca acinosa* polysaccharides I (PAP-I) was studied for the anti-tumor activities and their effects on the tumor necrosis factor (TNF) induction and peritoneal macrophage’s immunological cytotoxicity. PAP-I was given intraperitoneal 5–20 mg kg^−1^. d^−1^ x 7 d to ICR mice as a priming agent with subsequent lipopolysaccharides (10 micrograms/mouse) intravenously for TNF production. The crystal violet staining assay was used for TNF activity by using L929 cells. In a dose-dependent manner, the TNF production priming activity was shown by PAP-I along with hepatosplenic hyperplasia. PAP-I-treated peritoneal macrophages at the concentration of 10 and 20 mg kg^−1^ showed 67% and 74% cytotoxicity, respectively, against Meth A cells at effector:target = 40:1. PAP-I-treated peritoneal macrophages at the concentration of 10 and 20 mg.kg^−1^ also prolonged the survival time of mice bearing ascites Meth A tumor from 21±4 to 32±10 and 38±8 d and solid Meth A tumor growth inhibited with inhibition rate of 28.5% and 55.7%, respectively. Macrophage activation and TNF induction were responsible for PAP-I anti-tumor activities (Zhang and Qian, 1993).

An *in vivo* research study was conducted by H.B. Wang et al., in which they studied the anti-tumor effectiveness of *Phytolacca acinosa* polysaccharides I (PAP-I) at various concentrations of 5, 10, 20, and 40 mg/kg administered at intervals of seven times per week, three times per week, and once per week in sarcoma-180-bearing mice. It was confirmed from the results that PAP-I reached its optimal anti-tumor efficiency at 10 mg/kg, three times/week. Untreated mice were used as the control. These findings showed that PAP-I’s anti-cancer activity might primarily stem from its stimulation of mouse macrophages by regulating IL-1, IL-6, and TNF production (Wang and Zheng, 1997).

### Abortifacient activity

#### Molluscicidal activity

In a research study by Yeung et al., *Phytolacca acinosa* fresh roots, leaves, and seeds extracted one by one with 0.9% saline, and then cold acetone was added to the supernatants. The resultant precipitates were dialyzed against distilled water and lyophilized to form acetone powders. Acetone-precipitated powders of roots, leaves, and seeds were then tested for the *in vivo* abortifacient activity. Mature female ICR mice weighing 25–35 g were housed with fertile males in order to test the acetone powders for mid-term abortifacient action. Day 1 of pregnancy (PD1) was indicated by the presence of a copulation plug the next morning. On PD12, intraperitoneal acetone powders (AP) of *Phytolacca acinosa* roots, fresh leaves, and fresh seeds were given at a concentration of 10 mg/kg, 20 mg/kg, and 40 mg/kg. On PD14, the animals underwent autopsy. The total number of uterine implantation sites was noted. They counted the number of live fetuses, dead fetuses (hearts that do not beat), and resorbed fetuses. When there were more dead fetuses than 50% of the total implantation sites, the animal was deemed aborted. Saline was used as a control. The abortifacient activity of the AP seed was shown at 10 mg/kg, while the AP root and the AP leaf showed abortifacient activity at 20 mg/kg and 40 mg/kg, respectively. This abortifacient activity was susceptible to destruction by heat and pepsin (Yeung et al., 1987).

A group of scientists studied the rhizospheric strain of *Phytolacca acinosa Roxb* for *in vitro* molluscicidal activity against *Oncomelania hupensis*. The molluscicidal ingredient was isolated from the diethyl ether polar fraction of *Phytolacca acinosa* recognized as gliotoxin, which was tested for molluscicidal activity at various concentrations of 0.01 mg/L, 0.02 mg/L, 0.04 mg/L, 0.06 mg/L, 0.08 mg/L, 0.1 mg/L, 0.2 mg/L, 0.4 mg/L, and 0.6 mg/L. Gliotoxin demonstrated strong molluscicidal action with 24 h LC_50_ values of 0.101 mg/L and LC_90_ values of 0.355 mg/L, 48 h LC_50_ values of 0.062 mg/L and LC_90_ values of 0.121 mg/L, and 72 h LC_50_ values of 0.022 mg/L and LC_90_ values of 0.066 mg/L. The molluscicidal activity was determined by checking the mortality of snails. Dechlorinated tap water was used as the negative control, while niclosamide aqueous (1 mg/L) was used as the positive control (Guo et al., 2011).

**FIGURE 6:**
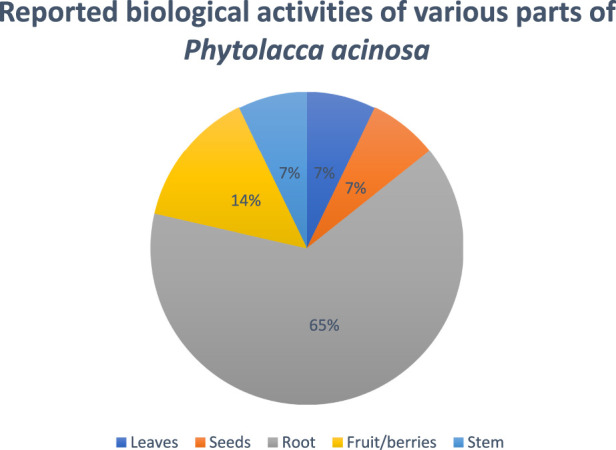
Percentage of biological activity studies associated with each part of *Phytolacca acinosa*, from all investigated articles.

In another study, G. Li et al., studied oncomelania-killing effects of the total saponins obtained from the roots of *Phytolacca acinosa* Roxb. After soaking for 24 h, the efficiency was found to be 95.6%, keeping the temperature at 28°C and the medication concentration at 125 mg/L (Li et al., 1998). Figure 6 showed the reported biological activities percentages.

#### Immunological activity

A study was conducted in which *Phytolacca acinosa* polysaccharides II (PAP-II) were investigated for lymphocyte proliferation and colony stimulating factor (CSF) production from splenocytes *in vitro*. The capacity of lymphocyte proliferation and CSF generation were assessed using the radioactivities of [3H] TdR uptake by bone marrow cells and lymphocytes, respectively. PAP-II was discovered to significantly increase Con A (1 and 2.8 micrograms. mL-1) and LPS (3, 10, and 30 micrograms. mL-1) induced lymphocyte proliferation at concentrations of 31–125 micrograms. mL-1 and significantly enhance splenocyte proliferation in a dose-dependent manner. Significant suppression of Con A-induced lymphocyte proliferation was seen as PAP-II concentrations increased. The current investigation confirmed that PAP-II from splenocytes can induce CSF. The ideal dosage was 100 micrograms per milliliter, and the best results were seen on day 5. These findings imply that PAP-II can improve hematopoiesis and immunological function (Wang et al., 1993a).

A research investigation examined *Phytolacca acinosa* polysaccharides I (PAP-I) for its effects on splenic lymphocyte proliferation and macrophage and splenic lymphocyte production of cytokines. Concanavalin A (Con A) was shown to stimulate only normal BALB/c and nude BALB/c splenic lymphocyte proliferation, but lipopolysaccharides (LPS) and PAP-I were found to significantly increase splenic lymphocyte proliferation of normal BALB/c, nude BALB/c, and NC mice *in vitro*. Moreover, PAP-I markedly boosted the mixed lymphocyte response and lymphocyte proliferation brought on by Con A or LPS. When given PAP-I for 5 days, splenic lymphocytes in normal BALB/c and nude BALB/c mice produced significantly more CSF, while NC mice’s lymphocytes did not. Interleukin-2 (IL-2) production from healthy mouse splenocytes was demonstrated to be greatly increased by PAP-I, and healthy mouse splenocytes were stimulated by Con A in a concentration-dependent manner. CSF activity was assessed using the supernatant of macrophages (M phi) that had received PAP treatment. The outcomes demonstrated that PAP-I dramatically increases M phi’s ability to secrete CSF activity on day 1. In addition, the supernatant contained a cytokine that worked in concert with recombinant murine granular-macrophage CSF (RMGM-CSF) to promote the proliferation of mice bone marrow cells, increasing splenic lymphocyte proliferation and IL-2 production with PAP-I, 5–50 mg.kg^−1^, intravenously. Our results suggest that both *in vivo* and *in vitro*, PAP-I can enhance immune function (Wang et al., 1993b).

A study conducted in which they investigated the *in vivo* effects of *Phytolacca acinosa* polysaccharides I (PEP-I) on mouse peritoneal macrophages’ immunologic cytotoxicity and their production of interleukin 1 (IL-1) and tumor necrosis factor (TNF). PEP-I 80 and 160 mg kg were administered intraperitoneally twice every 4 days. Both doses were discovered to significantly increase the cytotoxicity of macrophages toward S180 sarcoma cells and L929 malignant transformed fibroblast cells. To generate TNF and IL-1, respectively, peritoneal activated macrophages were treated with LPS for 2 and 24 h. The cytotoxicity of TNF and IL-1 against L929 cells was assessed using a co-stimulated absorbance assay of the enzymatic reaction and thymocyte proliferation. On day 8, it was discovered that TNF production was at its best. TNF and IL-I levels both significantly increased. These findings imply that TNF and IL-1 production by PEP-I-primed macrophages is tightly correlated with their cytotoxicity (Zhang et al., 1990).

#### Anti-microbial activity

A study conducted in which they studied the anti-microbial activity of different fractions of *Phytolacca acinosa*. Petroleum ether, ethyl acetate, butanol, and water, four different polarity extracts, were prepared from the root and stem parts of *Phytolacca acinosa* Roxb., respectively, to investigate the anti-microbial activities of fractions with different polarities and parts extracted from *Phytolacca acinosa* Roxb. Anti-microbial activities were tested against *Escherichia coli, Staphylococcus aureus, Vibrio parahaemolyticus*, and *Bacillus megaterium*. The findings revealed that the butanol extracts from the roots to *Bacillus megaterium* and *Vibrio parahaemolyticus* and the water extracts from the stem to *Vibrio parahaemolyticus* both have inhibitory zones with diameters of over 10 mm. The study also discovered that the best anti-bacterial fraction predominantly occurred in the root portion and that the best anti-microbial fraction predominated in high-polarity extracts, such as water and butanol (GuoDong et al., 2010). Compounds and extract of *Phytolacca acinosa* with pharmacological activities are shown in Table 3.

**TABLE 3:** shows the pharmacological activity of compounds and extracts of *Phytolacca acinosa.*

Compound/extract/fraction	Pharmacological activity	References
Phytolasides A and B	Anti-proliferation activity	Li et al. (2022)
Esculentoside B, cochliophilin B, and 6-methoxy-7-hydroxy flavone	Cytotoxic activity	Ma et al. (2017), Ma et al. (2019)
*Phytolacca acinosa* polysaccharides I	Anti-tumor activity	Wang and Zheng (1997), Zhang and Qian (1993)
*Phytolacca acinosa* fresh roots, leaves, and seeds extract	Abortifacient activity	Yeung et al. (1987)
Rhizospheric strain of *Phytolacca acinosa Roxb*	Molluscicidal activity	Guo et al. (2011)
Saponins from Phytolacca acinosa root extract	Molluscicidal activity	Li et al. (1998)
*Phytolacca acinosa* polysaccharides II	Immunological activity	Wang et al. (1993a)
*Phytolacca acinosa* polysaccharides I	Immunological activity	Wang et al. (1993b), Zhang et al. (1990)
Petroleum ether, ethyl acetate, butanol, and water fractions of *Phytolacca acinosa*	Anti-microbial activity	GuoDong et al. (2010)
Hydro-alcoholic root extract of *Phytolacca acinosa*	Antioxidant activity	Manzoor et al. (2017)
Polysaccharides from the root of *Phytolacca acinosa*	Antioxidant activity	Jing et al. (2010)
Esculentoside B	Anti-inflammatory activity	Abekura et al. (2019)
Acinospesigenin-A, -B, and -C	Anti-edemic activity	Koul et al. (2003)

#### Antioxidant activity

A study was undertaken to assess *in vitro* antioxidant properties of the hydro-alcoholic root extracts of *Phytolacca acinosa* and *Inula racemosa*. Roots that had been pulverized were used to prepare the hydro-alcohol extract. As compared to conventional ascorbic acid, the hydro-alcoholic root extracts of both plants demonstrated reducing power, DPPH activity, and hydrogen peroxide scavenging activity. The antioxidant activity of *Phytolacca acinosa* was also higher than that of the *Inula racemosa* root extract (Manzoor et al., 2017).

In another study, researchers isolated the polysaccharides from the root of *Phytolacca acinosa* Roxb, measured their quantity, and investigated the polysaccharides’ *in vitro* antioxidant activity. The *Phytolacca acinosa* Roxb root’s polysaccharides were prepared by degreasing with petroleum ether, extracting with hot water, decolorizing, precipitating with ethanol, and eliminating protein with trichloroacetic acid (TDA). The content of protein and polysaccharide in crude polysaccharide was determined using the Coomassie brilliant blue method and the anthrone-sulfuric acid method, respectively. The antioxidant activity of polysaccharides from the root of *Phytolacca acinosa* Roxb has been assessed by scavenging superoxide anion (O2^-^), hydroxyl free radical (^.^OH), and β-carotene-linoleic acid techniques using ascorbic acid (Vc) as a comparison sample. Analytical tests revealed that the pure sample had 92.01% polysaccharide, while the crude polysaccharide from the root of *Phytolacca acinosa* Roxb comprised 60.84% polysaccharide and 10.82% protein. Their antioxidant activity was determined by scavenging superoxide anion (O_2_^−^.), hydroxyl free radical (.OH), and β-carotene-linoleic acid methods. In terms of scavenging OH, the IC_50_ values for crude polysaccharide, pure polysaccharide, and Vc were 1.26 mg/mL, 4.78 mg/mL, and 0.224 mg/mL, respectively, whereas in terms of scavenging O_2_^−,^ IC_50_ values were 1.91 mg/mL, 2.28 mg/mL, and 0.123 mg/mL, respectively, while in terms of β-carotene-linoleic acid system inhibition, the IC_50_ values were 0.471 mg/mL, 0.692 mg/mL, and 0.379 mg/mL, respectively (Jing et al., 2010). Figure 6 shows the ratio of the number of studies (%) to the percentage of biological activity studies associated with each part of *Phytolacca acinosa*.

#### Anti-edemic activity

Another study isolated three new triterpenoids named as acinospesigenin-A, -B, and -C from the berries of *Phytolacca acinosa*. The anti-edemic activity of compounds was determined by the carrageenan-induced edema by measuring paw volume. These compounds showed anti-edemic activity (LD_50_ 10–15 mg/kg mass) in albino rats by inhibiting the serotonin, histamine, as well as cyclooxygenase enzyme activity, leading to inhibition of prostaglandin synthesis (Koul et al., 2003).

## Conclusion

*Phytolacca acinosa* is a significant medicinal plant with promising therapeutic potentials. It is a rich source of various phytochemical constituents including glycosides, flavones, terpenoids, saponins, and olenane derivatives. Some of the reported traditional uses of *Phytolacca acinosa* were supported by earlier pharmacological studies that were conducted on the plant. It is evident from the literature that this plant possesses strong antioxidant, cytotoxic, and anti-tumor activities, which make it a favorable candidate for further investigation of isolated metabolites for its anti-cancerous potential. Further scientific research is necessary to substantiate the stated traditional uses of *Phytolacca acinosa* because many of these uses lack supporting scientific proof. Nevertheless, only a small number of the identified bioactive metabolites from *Phytolacca acinosa* were tested for biological activity. Consequently, in order to completely demonstrate its therapeutic potentials and understand its intricate pharmacological action mechanisms, additional pre-clinical research on phytochemicals and other compounds may be conducted in the future. The safety profile of *Phytolacca acinosa* is also not well known.

## Limitations

In this review, we focused on articles related to traditional uses, phytochemistry, and pharmacological activities of *Phytolacca acinosa*. Articles focusing on other than traditional uses, pharmacological activities, and phytochemistry of *Phytolacca acinosa* were not included.

## Future perspective

Numerous metabolites have been isolated from *Phytolacca acinosa,* which possess biological activities; still a significant portion of intricate natural compound mixes remain unexplored chemically, offering a valuable resource for future scientific study. To ascertain the clinical significance of such compounds and to establish a reliable association with *in vitro* efficacy data, *in vivo* research in animal models may be conducted in the future. Compound structural alterations to enhance pharmacokinetics and pharmacodynamics, as well as evaluations of the structure–activity relationship, should be included in future research.

Further research should be conducted to elucidate the mechanism underlying these compounds’ biological activity and identify other pathways that could be targeted. Therefore, in order for these metabolites to be recognized as biomedical agents, more study is needed, particularly *in vivo* studies.
